# Functional and structural characterization of a novel putative cysteine protease cell wall-modifying multi-domain enzyme selected from a microbial metagenome

**DOI:** 10.1038/srep38031

**Published:** 2016-12-09

**Authors:** Muhammad Faheem, Diogo Martins-de-Sa, Julia F. D. Vidal, Alice C. M. Álvares, José Brandão-Neto, Louise E. Bird, Mark D. Tully, Frank von Delft, Betulia M. Souto, Betania F. Quirino, Sonia M. Freitas, João Alexandre R. G. Barbosa

**Affiliations:** 1 Laboratório de Biofísica Molecular, Departamento de Biologia Celular, Universidade de Brasília, Brasília, DF, 70910-900, Brazil; 2 Programa de Pós Graduação em Ciências Genômicas e Biotecnologia, Universidade Católica de Brasília, Brasília, DF, Brazil; 3 Diamond Light Source Ltd, Harwell Science and Innovation Campus, Didcot, OX11 0QX, England; 4 OPPF-UK, Research Complex at Harwell, Rutherford Appleton Laboratory, Oxford, OX11 0FA, United Kingdom; 5 Structural Genomics Consortium, Nuffield Department of Medicine, University of Oxford, Roosevelt Drive, Oxford, OX3 7DQ, UK; 6 Department of Biochemistry, University of Johannesburg, Auckland Park, 2006, South Africa; 7 Embrapa Agroenergia, Parque Estação Biológica - PqEB s/n°, Brasília, DF, 70770-901, Brazil

## Abstract

A current metagenomics focus is to interpret and transform collected genomic data into biological information. By combining structural, functional and genomic data we have assessed a novel bacterial protein selected from a carbohydrate-related activity screen in a microbial metagenomic library from *Capra hircus* (domestic goat) gut. This uncharacterized protein was predicted as a bacterial cell wall-modifying enzyme (CWME) and shown to contain four domains: an N-terminal, a cysteine protease, a peptidoglycan-binding and an SH3 bacterial domain. We successfully cloned, expressed and purified this putative cysteine protease (PCP), which presented autoproteolytic activity and inhibition by protease inhibitors. We observed cell wall hydrolytic activity and ampicillin binding capacity, a characteristic of most bacterial CWME. Fluorimetric binding analysis yielded a *K*_*b*_ of 1.8 × 10^5^ M^−1^ for ampicillin. Small-angle X-ray scattering (SAXS) showed a maximum particle dimension of 95 Å with a real-space R_g_ of 28.35 Å. The elongated molecular envelope corroborates the dynamic light scattering (DLS) estimated size. Furthermore, homology modeling and SAXS allowed the construction of a model that explains the stability and secondary structural changes observed by circular dichroism (CD). In short, we report a novel cell wall-modifying autoproteolytic PCP with insight into its biochemical, biophysical and structural features.

In the past decade, metagenomics has been utilized as a powerful technology for the discovery of novel enzymes and other valuable biomolecules produced by non-cultivated microbes^1^,^2^. The majority of the research using this technology aims to demonstrate the distribution of genes in a specific environment. This includes the function assignment of putative proteins via sequence homology or activity-based assays^3^,^4^. New enzymes have been isolated from metagenomic libraries constructed from various environments, many with potential for biotechnological and industrial applications^5^,^6^,^7^,^8^,^9^,^10^. Amongst enzymes, amidases and peptidases/proteases are especially important in industry^11^,^12^. A common substrate for these two groups of enzymes is the peptidoglycan present solely in bacterial cell walls^13^.

Peptidoglycan (PG) is a rigid biopolymer composed of alternating *N-*acetylglucosamine (NAG) and *N-*acetylmuramate (NAM) units linked by 1–4 glycosydic bonds between the two hexoses. Short peptides, containing both canonical L-amino acids and unusual D-amino acids, link the NAM units of the glycan chain. These peptides are synthesized via a ribosome-independent mechanism and hold together the glycan chains, giving the cell wall rigidity^13^. Biosynthesis of PG is carried out by a variety of conserved enzymes, such as racemases (which generate D-amino acids), glycosyl transferases (which form the hexose polymers), and peptyltransferases and transpeptidases (which form interpeptide linkages)^14^,^15^. The cell wall must go through reorganization during vegetative growth, development and cell division, which requires enzymes that hydrolyze various linkages in PG^13^,^16^,^17^. These enzymes include peptidases that cleave the cross-linking peptides, and glycosidases, such as lysozymes, that degrade the polysaccharide backbone^18^. In the case of bacteriophages, these enzymes can work as antimicrobial agents by hydrolyzing the host cell wall^19^.

A ubiquitous superfamily of cysteine, histidine-dependent amidohydrolase/peptidase (CHAP) was shown to be involved in cell-wall hydrolysis^13^,^20^,^21^. The CHAP superfamily shows no sequence similarity to other peptidase superfamilies, although the arrangement of catalytic residues, with respect to conserved secondary elements, is the same as that of several other peptidase superfamilies. In these cases, a catalytic cysteine at the amino terminus of a helix is packed against a core three-stranded β-sheet, where the second and third strands bear a catalytic histidine and its orienting polar partner^22^. Cysteine proteases (CPs) comprise a total of 108 different families^23^ and the catalytic residues can be ordered either Cys-His or His-Cys. In all the cysteine proteases, the Cys residue acts as the nucleophile agent, whereas the His residue acts as the general base for proton shuttling^24^. CPs are responsible for several biological processes including degradation of peptides and proteins^22^. Similarly, the biochemical functions of cell wall cysteine peptidases are known and structural information is also available^25^,^26^. A variety of cysteine proteases are synthesized as precursors that have a pro-domain and a mature (catalytic) domain. In some cases, a carboxyl terminal extension may also be present. The pro-domain has evolved diverse and independent functions, including acting as: an intramolecular chaperone to assist in protein folding; an endogenous inhibitor to regulate protease activity; and as a signal protein that targets the protease to its intracellular destination^27^. A number of these precursor cysteine proteases have autoproteolytic activity and are capable of cleaving and releasing their own functional domain or activating the proteolytic activity^28^,^29^,^30^,^31^.

The CHAP domain is also found in a wide range of protein architectures and is commonly associated with the bacterial type SH3 domains^32^,^33^,^34^,^35^. The *Staphylococcus aureus* autolysin LytA and other autolysins combine the CHAP domain with several families of amidases, forming bi-functional enzymes with multiple PG hydrolytic activities^20^,^21^. At least three types of unrelated amidase domains have been reported in proteins containing the CHAP domain, suggesting that CHAP domains have associated with amidase domains independently several times. These observations indicate that occurrence of multiple amidases within a single polypeptide chain is functionally important to provide tightly regulated cleavage of PG substrates^20^,^36^,^37^,^38^,^39^.

In 1994, Ghuysen *et al*. showed that PG hydrolases expressed by *Clostridia* and *Bacillus* strains had small conserved sequences that were signatures of proteins involved in cell wall binding^40^. These signatures are now indicators of PG-binding domains (PGBD), which are commonly found in the Protein Data Bank associated with cell wall degradation enzymes^41^,^42^,^43^.

Here we present a novel putative cysteine protease (PCP) selected from the metagenome of *Capra hircus* (Chi) rumen, hereinafter denoted as PCP. This novel protein carries an uncharacterized N-terminal domain, a cysteine protease/CHAP domain, a PG binding domain and a bacterial SH3 domain. The purified protease shows cell-wall hydrolytic activity and undergoes sequential autoproteolytic cleavage. Fluorescence spectroscopic analysis showed that PCP has ampicillin binding capacity. Circular dichroism spectroscopy revealed that the protein preserves its secondary structure under temperatures ranging from 25 °C to 95 °C. Solution state small-angle X-ray scattering (SAXS) studies of the protein enabled construction of a low-resolution, three-dimensional homology model of PCP.

## Results and Discussion

### Protein production and purification

Protein expression was performed at two different temperatures (28 °C and 37 °C), using two different concentrations of IPTG (0.5 mM and 1.0 mM). Expression was monitored at 1 hour intervals, up to 6 hours, and an overnight expression sample was also obtained. Optimum recombinant protein expression was obtained with 1 mM IPTG at 37 °C after 6 hours incubation (Fig. S1A in the supplementary information). PCP was purified through Ni-affinity chromatography after elution with 180 mM imidazole followed by size-exclusion chromatography (SEC). It presented a size of ~38 kDa after purification (Fig. S1B).

### Protein homogeneity and molecular weight

Dynamic light-scattering measurements were performed at a concentration of 13.2 μM of PCP in a 150 mM NaCl and 25 mM NaH_2_PO_4_ buffer at pH 8.5 and 25 °C; results indicated a particle with a hydrodynamic diameter of 6.87 nm and an estimated molecular weight (MW) of 53.1 ± 6.6 kDa (Fig. S2 in the supplementary information). The polydispersity index of these measurements was 14.9% and accounted for 99.3% of the particles present in the cuvette, indicating a pure and monodisperse protein sample^44^. The discrepancy between the theoretical molecular weight of PCP and the value encountered by DLS, respectively 37.9 and 53.1 kDa, may be explained if the shape of the protein is different from a sphere, since that is the expected shape used in the calculations. A rod-shaped protein would lead to an offset of higher molecular weight assignments. This is further corroborated by data from SEC-MALS analysis (size-exclusion chromatography with multi-angle light scattering), in which the MW of PCP was estimated to be between 39.8 and 42.3 kDa (Fig. S3). These data indicate that the particles are most likely present in a monomeric form of the protein in the given conditions.

### Protein secondary structure and stability

**Figure 1:**
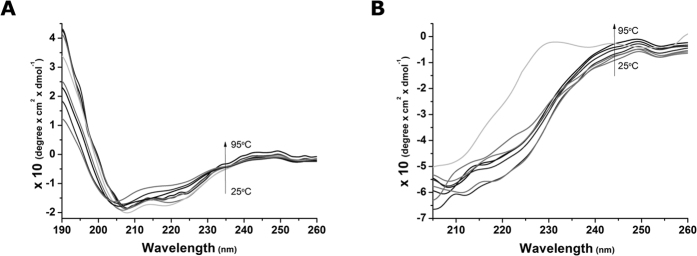
Circular dichroism spectra of PCP in TRIS-HCl with and without DTT as a function of temperature. (**A**) CD spectra obtained for the same sample at 8 temperatures ranging from 25 to 95 °C, in 10 °C steps, as represented by each curve. A characteristic α-helical profile is depicted, with minimums in 208 and 222 nm. (**B**) The same conditions were analyzed in the presence of 2 mM DTT. PCP secondary structure content was significantly altered by addition of DTT and even more so after subsequent heating.

The secondary structure profile of PCP was determined through circular dichroism (CD) spectroscopy. The far-UV CD spectrum obtained in the absence of DTT is characteristic of proteins that present mostly α-helices as secondary structures, with minimums at 208 and 222 nm and a positive band at 190 nm (Fig. 1A). The protein’s stability was assessed in temperatures ranging from 25 °C to 95 °C. Throughout this temperature range, only a small change in the secondary structure of PCP was observed: a small decrease in the amount of helical content alongside an increase in the β-sheet (Fig. 1A; Table 1). The considerable preservation of the secondary structure at very high temperatures led to the belief that the seven cysteine residues might participate in structure stabilization by means of disulfide bridges. Thus, the experiment was repeated in the presence of DTT and PCP lost most of its helical characteristic and no signal below 200 nm was recorded due to the high ratio of signal/noise (Fig. 1B). The secondary structure contents were estimated after deconvolution^45^ of the spectra in the presence and absence of DTT, as shown in Table 1.

### Sequence analysis and homology modeling

**Figure 2:**
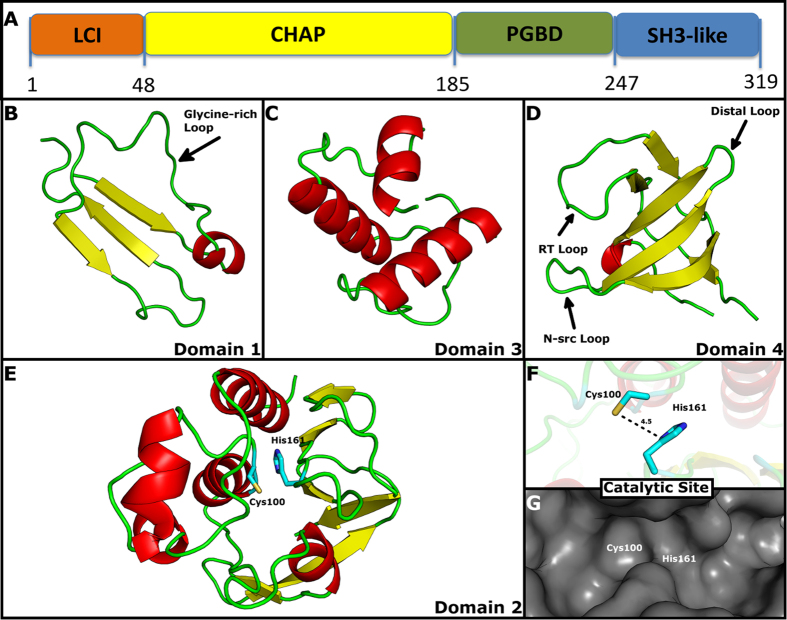
Cartoon representations of homology models of individual PCP domains. Alpha helices are colored in red and beta strands in yellow. (**A**) Domain 1 (residues 1-48), N-terminal domain is depicted in orange; Domain 2 (residues 49-185), the catalytic CHAP domain, in yellow; Domain 3 (residues 186-247), the peptidoglycan binding domain, in green; and Domain 4 (residues 248-319), the SH3 domain, in blue. (**B**) The N-terminal Domain 1 presenting an LCI fold. The arrow points to the glycine-rich loop observed in this fold. (**C**) Domain 3 presenting three alpha helices organized in a conserved PGBD fold. (**D**) The C-terminal Domain 4 presenting an SH3 conserved fold with its functional loops emphasized. (**E**) Domain 2 presenting the conserved structural features of a catalytic CHAP domain with a six-strand beta sheet (located to the right) packed against a group of alpha helices (located to the left). The catalytic residues, Cys100 and His161, are also shown. (**F**) Close-up on the catalytic site residues of Domain 2 and their respective distances. (**G**) Surface of the Domain 2 catalytic pocket showing the substrate cleft and catalytic residues’ positions.

The BLAST multiple sequence alignment indicated that PCP bears two conserved domains: a peptidoglycan-binding domain and a C-terminal SH3 domain. The Interpro server, on the other hand, classified PCP as bearing three conserved domains: the same two indicated by BLAST and an additional CHAP domain at the N terminus (Fig. 2A). Although most of the sequence was assigned to these three domains, the first 50 N-terminal residues were clearly not homologous to CHAP domains. Further investigation showed that this region, in fact, bore a hitherto undescribed domain. This fourth domain presents an LCI domain-like fold (Fig. 2A) and its identification is described in the supplementary information. The complete PCP homology model presents 23.8, 22.3 and 53.9%, of α-helices, β-sheets and random coils, respectively, and is in excellent agreement with the secondary structure content found in the CD experiments (Table S1 in the supplementary information).

### Domain 1

**Figure 3:**
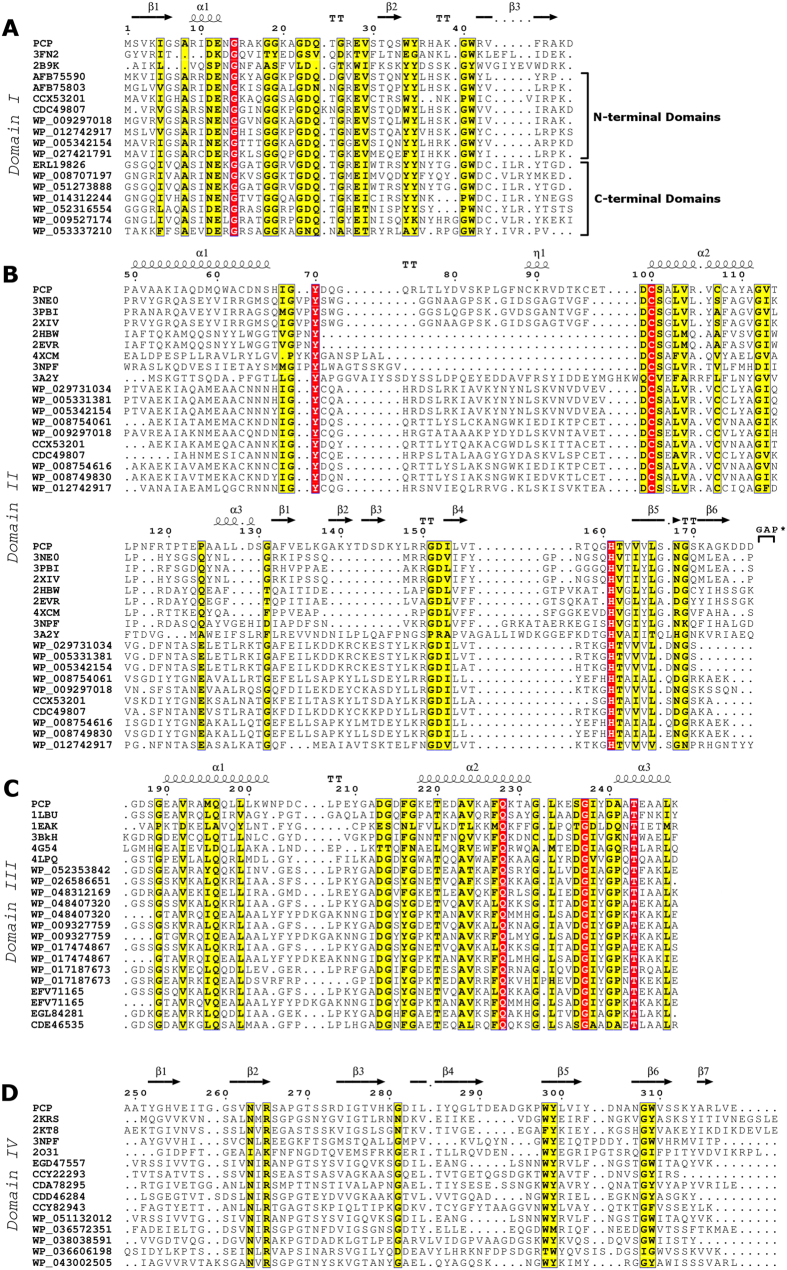
Sequence analysis of PCP domains. Secondary structures and residue numbering correspond to the PCP homology model and sequence, respectively. (**A**) Domain 1 sequence alignment to homologous sequences and PDB structures*. Homologous sequences found at the C-terminus or N-terminus of catalytic domains are grouped and identified in the insert. (**B**) Domain 2 sequence alignment to homologous sequences and PDB structures*. Conserved catalytic Cys100 and His161 residues are highlighted in red. At the end of this domain there are 8 residues missing referred to as GAP: _178_PVTDALKR_185_. (**C**) Domain 3 sequence alignment to homologous sequences and PDB structures*. Conserved PBD signature sequence repeat can be seen at position Asp213-Thr230 and Asp236-Thr243 (in PCP residue 236 is a Ser). (**D**) Domain 4 sequence alignment to homologous sequences and PDB structures*. *All PDB structures are identified by their four-character accession codes (ex. 3FN2, 3NE0, 1LBU, 2KRS etc). This figure was produced using the ESPript online server (http://espript.ibcp.fr)^89^.

Domain 1 (D1) is present at the N terminus and ranges from residues 1 to 48. It presents an LCI domain-like fold constituting a β-sheet of three antiparallel β-strands in a β_1_β_3_β_2_ topology (Fig. 2B). The LCI domain (Pfam PF12197, InterPro IPR020976) is approximately 40 amino acids in length, commonly found in bacteria of the *Bacillus cereus* group and functionally related to antimicrobial activity. The LCI fold (Fig. S4) is based on a single PDB entry, accession 2B9K, although it has been found in other proteins such as the C-terminal region of a putative sensor histidine kinase domain (PDB accession 3FN2; Fig. S4). Sequence alignment between D1-homologues shows the presence of a highly conserved glycine-rich motif corresponding to ExGxxxGGxxGDQxGxE, suggesting structural and functional conservation (Fig. 3A). BLASTp analysis of this motif shows that a variety of proteins carry this sequence, many of which are unrelated to CHAP domains (e.g. WP_054337895).

### Domain 2

Domain 2 (D2) is composed of residues 49 to 185; it is a catalytic domain belonging to the superfamily of cysteine, histidine-dependent amidohydrolases/peptidases (CHAP). The domains from the CHAP superfamily consist of ~110–140 residues with two strictly conserved residues, a cysteine and histidine that forms a catalytic Cys-His dyad. In PCP, the dyad corresponds to Cys100-His161 (Figs 2E and 3B). Proteins presenting these domains are highly modular, with multiple components often fused to form a multifunctional protein^20^,^21^,^25^. The CHAP domains are mainly involved in cell-wall hydrolysis and the components fused to it often depict and synergize with this function. Examples of frequent components include an N-terminal signal peptide, a MurNAc amidase, and one or multiple targeting domains, such as the LysM domain, the peptydoglycan binding domain (PGBD), the choline binding domain (CGD), and the bacterial SH3b domain^34^. Recently, the crystal structure of a CHAP domain has been characterized as a cellulosome-related module, indicating that this family of cysteine peptidases might modulate processes other than cell-wall hydrolysis^46^. CHAP domains have been unified under the peptidase families C40 and C51 in the MEROPS database^23^, under Pfam^47^ domains NlpC/P60 (PF00877) and CHAP (PF05257), and under the COG database^48^ entries COG0791 ‘cell-wall-associated hydrolases (invasion-associated proteins)’ and COG3942 ‘surface antigen’. The overall fold of domain 2 consists of a tight interface between an N-terminal α-helical sub-domain and a C-terminal sub-domain comprised of six antiparallel β-strands in a β_1_β_2_β_6_β_3_β_4_β_5_ topology (Fig. 2D). The fold resembles that of the papain family of cysteine proteases, where the interface bears the conserved catalytic cysteine (helix α_2_) and histidine (strand β_4_). It is likely that all members of the CHAP superfamily share the proposed nucleophile-attack mechanism where the conserved cysteine residue acts as the catalytic nucleophile^20^,^49^. All functionally characterized enzymes of the CHAP superfamily—that is, *N-*acetylmuramoyl-L-alanine amidases (autolysins), glutathionylspermidine amidases, γ-D-glutamyl-L-diamino acid endopeptidases and γ-D,L-polyglutamate depolymerase—are γ-glutamyl D,L-endopeptidades that hydrolyze diverse substrates containing the γ-glutamyl moiety. A third conserved residue, Tyr70, is important in the formation of a putative oxyanion hole that stabilizes negative charge of the substrate-enzyme intermediate^50^.

CHAP superfamily members also share structural similarities that cannot be detected at the sequence level^46^. Beyond the catalytic core, the structures are highly divergent through deletions and insertions of structural elements. While the catalytic core and interface resemble that of other papain-like proteases, the quantity of residues between the helix bearing the catalytic cysteine and the core β-sheet is similar to that seen in the primary structure of transglutaminases and NH_2_-aminotransferases, suggesting that it lacks the large insert seen in several papain-like proteases. This type of difference can explain why sequence-structure threading algorithms fail to recover any significant hit to papain-like proteases. Other types of differences can be seen in several structures in the PDB. Considering the D2-homologues in the PDB (4HZ9, 2EVR, 2HBW, 4XCM, 3I86, 2XIV, 3NE0, 3PBI and 3A2Y), the C-terminal β-sheet sub-domain can be organized into two topologies and then further into two distinct structural groups. The first topology corresponds to that of PCP and PDB accession 3A2Y: β_1_β_2_β_6_β_3_β_4_β_5_ (topology 1; Fig. S5C and S5D in the supplementary information). The second topology covers the remaining homologue structures and portrays a β_1_β_6_β_2_β_3_β_4_β_5_ sheet (topology 2; Fig. S5A and S5B). In the case of D2-homologues, the presence or absence of a secondary structure element between strands β_5_ and β_6_ is an evidence of divergence in members of the CHAP superfamily (Fig. S5). D2-homologues portraying topology 1 either have a simple loop (3I86, 2XIV, 3NE0 and 3PBI) or an α-helix (4HZ9, 2EVR, 2HBW and 4XCM) between β_5_ and β_6_. The same happens for topology 2, where a simple loop (PCP) or a two-strand β-sheet is present (3A2Y).

### Domain 3

A peptidoglycan-binding domain (PGBD) spans residues 186 through 247. The PGBD, as well as related domains that share the same structure, may have a general PG binding function that allow protein bearing it to increase their concentration on the bacterial cell wall. Its core structure consists of a closed, three-helical bundle with a left-handed twist. The PGBD from PCP presents the conserved cell wall-binding residues that have been previously reported^41^ (Fig. 3C). A variety of enzymes involved in bacterial cell wall degradation carry this domain at the N or C terminus^51^,^52^,^53^. Like PCP, many of the proteins bearing this domain are yet uncharacterized, but some have been grouped by MEROPS into the M15A subfamily of metallopeptidases, which belong to the M15 peptidase family. A number of the proteins belonging to subfamily M15A are non-peptidase homologues, as they either have been found experimentally to be without peptidase activity or lack amino acid residues believed to be essential for catalytic activity^23^.

### Domain 4

The fourth domain is recognized as an SH3 domain comprising residues 248 to 319. The SH3 domains are small protein modules containing approximately 50 amino acid residues (Figs 2D and 3D). SH3 stands for Src homology 3 domain and was first found in the Src family of tyrosine kinases. The classical SH3 domain is found in proteins that interact with other proteins, where it mediates the assembly of specific protein complexes, typically via binding to proline-rich peptides in their binding partner; they were the first modular binding domains found to bind constitutively to their partners without the need of post-translational modifications^54^. While it is safe to assume that most SH3-binding epitopes of proteins bear the consensus short linear motif PxxP (where x are aliphatic residues)^55^, some exceptions have been reported: the Pix SH3-binding site in PAK kinases (PPVIAPRPETKS)^56^, a class of enzymes targeted by small GTP binding proteins and implicated in a wide range of biological activities; the SH3-binding consensus of Eps8 (PxxDY)^57^, a substrate of receptor and non-receptor tyrosine kinases; and the Hbp SH3-binding sites on UBPY (Px(V/I)(D/N)RxxKP)^58^, a deubiquitinating enzyme. The function of SH3 domains is not entirely understood, but they may mediate many diverse processes by increasing local concentration of proteins, altering their subcellular location and mediating the assembly of large multiprotein complexes^54^. In the case of rumen enzymes, such as PCP, SH3 domains may mediate binding to plant substrates via proline-rich proteins encountered in plant cell walls^59^. What is clear is that the surface of the SH3 domain bears a relatively flat hydrophobic ligand-binding interface, which consists of three shallow grooves defined by conserved aromatic residues (Fig. 2D)^35^. All SH3 domains consist of two small β-sheets, totaling five or seven β-strands, which are packed approximately perpendicular against each other. Three variable loops can be identified when SH3 domains are compared; these are termed the RT, N-Src, and distal loops. The RT and N-Src loops are on the ligand-binding face of the domain and can modulate binding, whereas the distal loop is on the opposite face and might interact with other regions of the same protein (Fig. 2D).

### Small angle X-ray scattering (SAXS)

**Figure 4:**
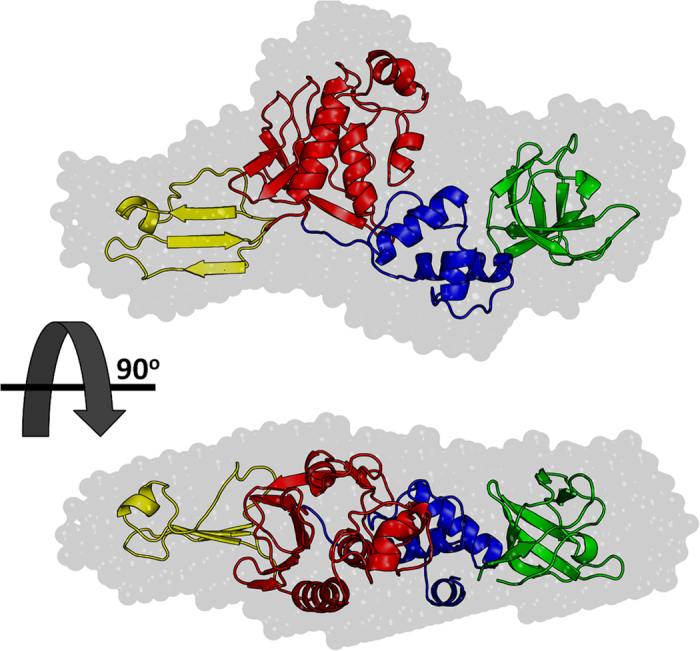
SAXS envelope fitted with the complete homology model of PCP. The images are rotated with respect to each other by 90 degrees on the longer axis. Individual homology models of each domain were manually fitted into SAXS envelope using the PyMOL software and then modeled together to produce a complete homology model of PCP. The complete model was analyzed with the SAXS envelope using the software SCATTER^88^.

No radiation damage was detected for the protein samples as the first and last frames of the exposure set were indistinguishable. A stable Guinier region from 0.01575 to 0.046 Å^−1^ (112 points) was observed estimating a reciprocal-space R_g_ of 28.8 Å for the PCP protein. Further analysis indicates a particle volume of 93,000 Å^3^ with a Porod-Debye exponent of 3.5. In addition, a dimensionless Kratky plot reveals a non-globular protein (Fig. 4). These parameters suggest the protein, in its thermodynamic state, is elongated with minor conformational flexibility. Indirect Fourier transform of the SAXS data (q_max_ 0.2 Å^−1^) to real-space indicates a maximum particle dimension (d_max_) of 95 Å with a real-space R_g_ 28.4 Å. The SAXS analysis indicates the sample was of sufficient quality for further *ab initio* analysis using DAMMIF to a q_max_ of 0.2 Å^−1^. The envelope generated from these data was used to fit the homology model with four domains with quite a good agreement (Fig. 4). Furthermore, the molecular weight of PCP was estimated from the SAXS data using the approach from Rambo and Tainer^60^. This approach yielded an estimated MW of 44.8 kDa which is similar to the estimate from SEC-MALS analysis (39.8 to 42.3 kDa). Altogether, these data indicate that PCP is a monomer in solution.

### Autoproteolysis assays

**Figure 5:**
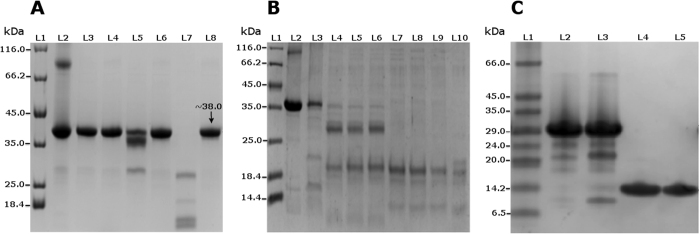
12% SDS PAGE showing recombinant PCP protein autoproteolytic assays. Figure 5 (**A**) Lane 1: Protein maker. Lane 2: Purified denatured protein at 95 °C as native control. Lane 3: Protein incubated at −21 °C showing no autoproteolysis. Lane 4: Protein incubated at −21 °C with protease inhibitors cocktail. Lane 5: Protein incubated at 4 °C showing partial autoproteolysis. Lane 6: Protein incubated at 4 °C with protease inhibitors cocktail with no autoproteolysis. Lane 7: Protein incubated at 25 °C showing complete autoproteolysis. Lane 8: Protein incubated at 25 °C with protease inhibitors cocktail showing that the autoproteolysis was inhibited. (**B**) Lane 1: Protein Marker. Lane 2: Purified denatured protein at 95 °C. Lane 3: through Lane 10: Purified protein incubated at 25 °C in different buffered pHs: 4.1, 4.8, 5.6, 6.1, 6.8, 7.2, 7.9 and 8.5, respectively. An increase in the autoproteolytic activity of PCP can be correlated with the increase in pH. (**C**) Lane 1: Protein marker; Lane 2: Purified construct of domains 1 through 3 (D13) incubated at 4 °C showing autoproteolytic activity; Lane 3: Purified D13 incubated at 25 °C and displaying autoproteolytic activity; Lane 4: Purified construct of domains 3 and 4 (D34) incubated at 4 °C and displaying no autoproteolysis; Lane 5: Purified D34 incubated at 25 °C and displaying no autoproteolysis.

PCP was incubated at different temperatures (−21 °C, 4 °C and 25 °C) to assess the autoproteolysis profile (Fig. 5A). These samples were analyzed after overnight incubation and autoproteolytic activity was observed at all the temperatures, except −21 °C. Autoproteolytic degradation at 25 °C was greater than at 4 °C. In order to observe complete proteolysis of the protein at different temperatures, PCP was incubated for 4 days (Fig. 5A). Inhibition of the autoproteolysis could be observed at all the temperatures when samples were incubated with a cocktail of protease inhibitors. Interestingly, PCP incubated with high concentration of imidazole showed no autoproteolytic activity (data not shown). Autoproteolysis of PCP increased as pH ranged from 4.1 to 8.5 (Fig. 5B). In Fig. 1C, D13 (construct carrying domains 1 through 3) shows clear autoproteolytic activity, while D34 (construct of domains 3 and 4) does not. The autoproteolysis band profile of purified D13 (28.5 kDa) incubated at 25 °C and 4 °C indicates that domain 3 (7.2 kDa) becomes separated from domains 1 + 2 (20.0 kDa). Autoproteolytic activity was higher at 25 °C than at 4 °C. No autoproteolysis was verified for purified D34 (14.6 kDa) incubated at 25 °C and 4 °C, even after incubation for one week (Fig. 5 C). Altogether these results show that domain 4 is not responsible for autoproteolysis and suggest that domain 2 holds this activity, as further corroborated by the homology model of the PCP.

### Cell wall hydrolysis

**Figure 6:**
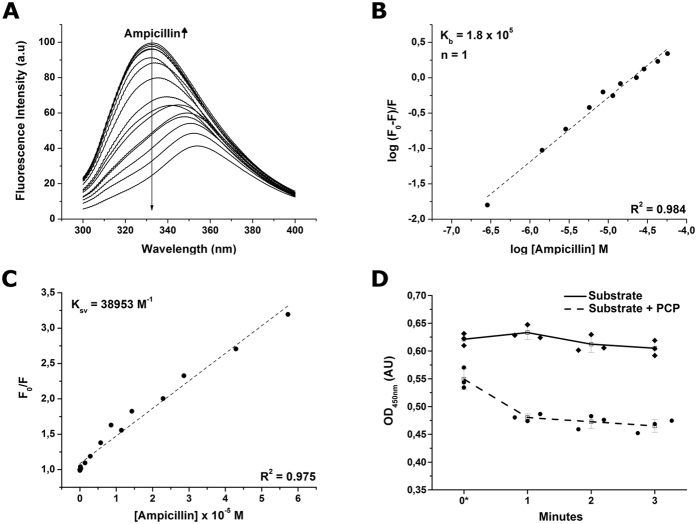
Fluorescence quenching spectroscopy of PCP in complex with ampicillin and results of a cell wall hydrolase assay. (**A**) Fluorescence emission spectra of the protein with increasing concentrations of the ampicillin (0.0029 to 57.0 μM) indicated by the thin arrow. A decrease in the fluorescence emission spectrum was observed as ampicillin concentration increased. (**B**) Double logarithm regression curve as a function of ampicillin concentration. Binding constant (K_b_) and number of binding sites (n) are also depicted. (**C**) The linear regression derived from Stern-Volmer approximation. The Stern-Volmer constant (K_SV_) is also depicted. (**D**) Cell wall hydrolase assay showing decrease in the OD_450nm_ due to PCP activity. Round dots and dashed line refer to the experiments in the presence of PCP while diamond-shaped dots and full line are the control experiments containing buffer without the enzyme. Error bars represent 1.5 times the standard deviations of a set of three replicates, as represented by three dots for every time measurement (when the 3 dots of one specific time overlapped, the dots were slightly separated along the time axis for clarity). The line connecting measurements passes through the mean average of each triplicate (unfilled squares). *Zero minutes represents the moment the experiment was first measured; however, there was a delay of approximately 2 minutes accounting for the time between adding the enzyme and setting up the experiment for its first measurement.

PCP incubation with cell wall suspension showed a decrease in the OD_450nm_ during the initial minutes of the reaction, indicating that PCP hydrolyzed the cell wall. The initial difference of 0.08 AU seen in the OD_450nm_ between control buffer and PCP at zero minutes can be attributed to the time elapsed between PCP addition to sample and setting up the experiment for the first measurement, indicating that hydrolysis starts immediately after addition of PCP to the substrate (Fig. 6D). This phenomenon was observed at t = 0 min in various repetitions of the experiment using different concentrations and pHs (data not shown).

### Fluorescence spectroscopy and ampicillin binding

The protein’s interaction with ampicillin was evaluated via fluorescence quenching by adding gradual amount of ampicillin while keeping the PCP concentration at 5.28 μM (0.2 mg.mL^−1^). An increase of the concentration of ampicillin caused a progressive decrease in fluorescence intensity and a red shift from 330 to 354 nm, as shown in Fig. 6A. These results are compatible with an ampicillin-PCP interaction leading to conformational changes promoting the complete exposure of tryptophan residues from buried to polar environment. The Stern-Volmer constant (*Ksv*) was calculated from the linear regression presented in Fig. 6B. The value of *Ksv* is five times larger in magnitude than those for diffusion-limited quenching of free tryptophan in water. This can be attributed to the static quenching process^61^ caused by the ampicillin-PCP complex formation before the excitation state. The fluorescence intensities at 330 nm were fitted according to equation 2 to obtain the binding constant of ampicillin-PCP complex. It was calculated assuming the equilibrium was reached between free and bound molecules that can bind independently to a set of equivalent sites in a single macromolecule (Fig. 6C). The *K*_*b*_ of 1.8 × 10^5^ M^−1^ allows to conclude that the ampicillin-PCP interaction is of high affinity.

## Conclusions

Metagenomics allows the study of the DNA of an entire population of microorganisms^62^. This technique has been utilized for the discovery of new proteins from metagenomic libraries that are screened for specific activities^6^. While exploring the metagenome of the gut microbiota of the *Capra hircus* we have found a novel cysteine protease (PCP). Sequence analysis of the PCP using different databases has indicated that it is a protein with a rare four-domain composition: an N-terminal LCI fold domain, a CHAP domain, PGBD domain and a C-terminal SH3 domain. Each domain was modeled based on its own conserved domain’s topology. The multi-domain structure was assembled under the guidance of an elongated SAXS envelope (d_max_  = 95 Å and R_g_ = 28.4 Å). This elongated model explains the higher molecular weight observed with DLS experiment and is further corroborated by its good agreement to the secondary structure content calculated from experimental CD data. For the first time, the LCI fold domain is being reported linked to CHAP, PGBD and SH3 domains and although its structure has been associated with antimicrobial activity, so far there are no publications about it. The CHAP domain possesses the conserved active site dyad, Cys100 and His161, along with other residues that shall participate in the active site. Besides Cys100, another six cysteines are present and some are in contact distance to foster disulphide bonds. This observation is corroborated by the CD measurements in the presence of DTT showing denaturation, especially the α-helical content where the cysteine residues are present. Similar to other reported cysteine proteases, the purified PCP has autoproteolytic activity as established by assays with and without protease inhibitors. This activity was higher at 25 °C and in the pH range 6.8–7.9, which is an optimum pH for cysteine protease activity. Furthermore, the expressed and purified construct of the first three domains (D13) showed autoproteolytic activity while another construct containing the last two domains (D34) did not, strengthening the idea that the CHAP domain 2 is the one responsible for this activity. Cell wall hydrolysis has also been observed for PCP in the first three minutes of assay incubation. Fluorescence experiments show a ampicillin binding to PCP with a high affinity *K*_*b*_ (1.8 × 10^5^ M^−1^), a feature encountered in other cell wall hydrolases.

Considering that PCP was selected from a metagenomic library containing huge DNA information of the *Capra hircus* gut, its role in such an environment could be diverse. Nonetheless, our study corroborates reports in literature in which proteins composed of CHAP and SH3 domains play major role in cell wall degradation, repair and expansion through peptydoglycan hydrolyses. The rumen microbiota breaks down cellulose and other polysaccharides, providing volatile fatty acids for the host and sugars for the symbionts. With increasing amounts of sugars, lactate producing bacteria grow and proliferate to the point where their exceeding biomass leaks to the abomasum and provides the ruminant with its main source of proteins. During this process the peptydoglycan network is dynamic and constantly broken down by enzymes to accommodate bacterial cell growth in a process known as cell wall turnover. As much as 50% of peptydoglycan is degraded during each generation and the turnover products are typically recovered and eventually recycled for *de novo* peptydoglycan biosynthesis in a process that involves multiple dedicated enzymes^63^. One can conclude that where there is sugar hydrolysis and uptake by bacteria, there must also be concomitant peptydoglycan enzymes acting to accommodate their growth and proliferation^39^. In this regard, the PGBD and SH3 domain from PCP may form a bridge between the bacterial cell wall and the cell wall of plant substrates via their respective binding to these organelles. This correlation is also strengthened by the discovery of a CHAP domain acting as module in a cellulosome^46^. Recently, Xu and coworker have shed a light on the differences between substrate specificity of one turnover and two recycling cysteine proteases^64^. However, none of their discoveries could be related to PCP (data not shown). Our data suggest that PCP is a unique enzyme, comprised of a previously uncharacterized catalytic domain that presents low identity (31% with 59% coverage) to the closest structure deposited in the PDB and two types of activity (autocatalytic and peptydoglycan hydrolysis). Furthermore, this catalytic domain is in module with three domains that have never been concomitantly described together, one of which was a hitherto unknown domain for the first time described and whose function is still unclear.

## Methods

### Gene selection and domains analysis

DNA sequences of the previously reported goat rumen (*Capra hircus*) microbiota metagenomic library^65^ were analyzed for open reading frames (ORFs) using ORF-finder (http://www.ncbi.nlm.nih.gov/gorf/gorf.html). The domain classification of the selected ORF was performed using InterPro blast^66^. The MEROPS database was used to perform a search for protease homology and possible active site amino acid residues^23^. The selected ORF was aligned to homologous sequences using BLAST program provided by NCBI. Multiple alignments with homologous proteins and conserved amino acid residues analysis, as well as the active site residues, was performed using ClustalW2 program (http://www.ebi.ac.uk/Tools/msa/clustalw2/).

### Homology modeling

To guide the comparative modeling, a secondary structure prediction of PCP was obtained from the servers PSIPRED^67^, Phyre 2.0^68^ and Jpred4^69^. A prediction of domain boundary was obtained from ThreaDom Online^70^, in which three domains were identified for PCP. Based on the consensus secondary structure prediction and domain boundary predictions, the PCP amino acid sequence was fragmented into four different parts which were submitted individually to four automated protein structure modeling servers: LOMETS^71^, SWISS-MODEL^72^, QUARK^73^ and M4T 3.0^74^. Tridimensional models comprising these fragments were generated using the homologous domains from protein databank (PDB) entries 2B9K, 3FN2, 3PBI, 2KRS, 2KT8, 2XIV, 4Q4G, 3A2Y, 3NPF, 3NE0, 2EVR, 1LBU, 4LPQ, 3BKH, 4G54, and 4KCA, and were further evaluated for quality in the RAMPAGE^75^ and ProSA-Web servers^76^. The best models and their respective original templates were selected as templates for subsequent modeling using the MODELLER v9.14 program^77^. The input alignment used by MODELLER was generated through the MUSCLE algorithm^78^ and utilized in various combinations to generate complete and truncated models of PCP. These models were analyzed in the QMEAN^79^ and Molprobity servers^80^. The best model was submitted to four refinement servers: ModRefiner^81^, KoBaMIN^82^, 3Drefine^83^ and Yasara^84^. The refinement outputs were reanalyzed by QMEAN and Molprobity to verify the improvement and the best-refined model was selected as the template for a new round of modeling using MODELLER. This process was repeated until the acquisition of a tridimensional model that corroborated the consensus secondary structure and domain boundary predictions possessed a Ramachandran plot with <1% of Φ and Ψ angles in disallowed positions, a QMEAN-score >0.6, and acceptable geometric parameters according to Molprobity (green, yellow and <2% red color representation). A detailed description of each step in this process is available in the supplementary information.

### Gene amplification and cloning

The selected ORF (PCP) was PCR amplified from the goat rumen metagenome library clone using the following primers: forward primer 5′ *CAT ATG* TTG GGA CAA ATC AGC ACA GCA G 3′, with restriction site *NdeI* shown in italics; and reverse primer 5′ *CTC GAG* TTC GAC CAG TCG GGC GTA CTT G 3′, with restriction site *XhoI* shown in italics. The PCR reaction was performed using 0.5-1 μg of the plasmid DNA^65^ carrying the ORF, 0.2 mM of dNTP mix, 0.25 mM of forward and reverse primers, 1 U/μL of platinum *Pfx* Taq polymerase (Invitrogen, USA), 1X Taq buffer (Invitrogen, USA) and nuclease-free water (MilliQ), in a total final PCR reaction volume of 50 μL. DNA amplification was performed in a thermal cycler for 35 cycles of denaturing at 94 °C for 30 seconds; annealing at 60 °C for 30 seconds; and extension at 68 °C for 90 seconds. The amplified PCR product was purified and cloned in a pGEM^®^-T vector supplied with the TA cloning kit (Promega), according to the manufacturer’s instructions. The resulting ligated product was transformed into *E. coli* Top10 cells. The recombinant pGEM^®^-T plasmid DNA was extracted from positive clones and digested with *NdeI* and *XhoI* restriction enzymes. The digested DNA was purified and subcloned into a *NdeI* and *XhoI* digested pET-28a vector. Plasmid constructs were prepared for domains 1 (D1) to 3 (D3), termed D13, for domains 3 and 4 (D4), named D34. D13 was PCR amplified with primers forward 5′ AGG AGA TAT ACC ATG TCC GTG AAG ATC GGC AGC GCG AG 3′ and reverse primer 5′ GTG ATG GTG ATG TTT GGT CGC CGC CTT CAG CGC CGC C 3′ and D34 with forward primer 5′ AAG TTC TGT TTC AGG GCC CGA TGT CCG TGA AGA TCG GCA GCG C 3′ and reverse primer 5′ATG GTC TAG AAA GCT TTA TTC GAC CAG TCG GGC GTA CTT GC 3′. PCR reaction was prepared using 25 μL 2 X Phusion Flash Master Mix (Thermoscientific, USA), 3 μL (10 μM) forward and reverse primers each, 2 μL (20 ng/μL) template plasmid DNA (pET28a) gene construct (described above), reaction volume was completed to 50 μL by adding 17 μL of nuclease free water (MilliQ). DNA amplification was performed in a thermal cycler with initial denaturation of 98 °C for 10 seconds; 35 cycles of denaturing at 98 °C for 10 seconds; annealing at 60 °C for 5 seconds; and extension at 72 °C for 120 seconds. PCR product was purified with AMPure XP Magnetic Beads (Beckman, USA). Purified PCR product for D13 (3 μL) was mixed with 1 μL (100 ng) of linearized pOPIN F vector (OPPF, UK) and D34 (3 μL) was mixed with 1 μL (100 ng) of linearized pOPIN E vector (OPPF, UK). Reaction volume was completed to 10 μL by adding 7 μL nuclease free water. This 10 μL of reaction mixture was mixed with dry-down In-Fusion reagent (Clontech, USA). The reaction mixture was incubated at 42 °C for 30 minutes. Reaction was stopped by adding 40 μL of TRIS-EDTA. 3 μL of this reagent mixture (ligated vector) was transformed in *E. coli* (OmniMacII) cells. Positive colonies were screened for recombinant plasmids by PCR amplification.

### Expression and purification

#### Full length PCP

The recombinant pET-28a plasmid was transformed into *E. coli* BL21 (DE3) pLysE cells. A single colony of the transformed bacteria was grown in 20 mL of Luria Bertini (LB) medium with 50 μg/mL of kanamycin and 37 μg/mL of chloramphenicol, and incubated at 37 °C overnight. The overnight grown cells culture was inoculated into 500 mL with the same antibiotics. This culture was grown in a shaking incubator at 37 °C and 220 rpm until the optical density at 600 nm (OD_600nm_) reached 0.6. Protein expression was induced with 1 M isopropyl-β-D-galactopyranoside (IPTG) and incubated for 6 hours at 37 °C. Cells were pelleted after 30 minutes at 7000 g and 4 °C. The pellet was resuspended into 20 mL of lysis buffer (300 mM NaCl, 50 mM NaH_2_PO_4_, pH 8.5) and sonicated on ice. The supernatant was collected after centrifugation at 7000 g for 30 minutes at 4 °C. The lysed supernatant (20 mL) was filtered (with a syringe filter of 0.45 μm) and applied to a 1 mL His Trap HP nickel column (GE Healthcare Life sciences, UK). Protein purification was performed in gradient purification using GE AKTA Purifier (GE Healthcare Life sciences, UK) with 300 mM NaCl, 50 mM NaH_2_PO_4_ and 500 mM imidazole buffer at pH 8.5. The UV absorption was monitored at 280 nm and the purified protein (8 mg/mL) was re-purified in a size-exclusion chromatography (Superdex 75, 100/300 GL) with buffer (150 mM NaCl, 25 mM NaH_2_PO_4_ pH 8.5).

#### PCP domains

The Plasmid DNA for D13 and D34 in pOPIN-E and pOPIN-F vectors respectively was transformed in *E. coli* Lemo21 (DE3). A single colony of transformed cells was first grown overnight in LB media supplemented with 50 μg/mL ampicillin and 38 μg/mL at 37 °C with 220 rpm shaking. 25 mL of overnight cultured cells were added to 1 L of overnight express instant TB media (Merck Millipore, USA) supplemented with 50 μg/mL ampicillin and 38 μg/mL at 37 °C with 220 rpm to an OD_600nm_ of 0.6. The growing cells were transferred to room temperature for 30 minutes, induced with 1 mM IPTG and incubated overnight at 25 °C with 220 rpm. Cells were harvested at 5000 g for 15 minutes at 4 °C and re-suspended in lysis buffer (50 mM NaH_2_PO_4_ pH 7.5, 300 mM NaCl, 10 mM Imidazole). Cells were disrupted with constant cell disruption system under pressure at 25 KPSI (CONSTANT SYSTEMS Ltd, UK) and centrifuged at 34000 g for 20 minutes at 4 °C. Supernatant was applied to 5 mL Ni Sepharose column (GE) and purified in gradient purification with buffer (50 mM NaH_2_PO_4_ pH 7.5, 300 mM NaCl, 500 mM Imidazole). D13 purified protein was re-purified on a Superdex 75 16/600 GL (GE) column equilibrated with purification buffer (150 mM NaCl, 20 mM NaH_2_PO_4_ pH 7.5). D34 purified protein was mixed with HRV 3 C protease to cleave N-terminal his-tag. Cleavage was performed in 3 kDa dialysis membrane in dialysis buffer (50 mM NaH_2_PO_4_ pH 7.5, 300 mM NaCl, 22 mM Imidazole, 1 mM TCEP) with continuous magnetic stirring overnight at 4 °C. Cleaved protein was re-purified on a Ni Sepharose column (GE) with the same overnight dialysis buffer. Column flow through was collected that has his-tag free protein. Purified protein was re-purified on a Superdex 75 16/600 GL (GE) column with purification buffer (150 mM NaCl, 20 mM NaH_2_PO_4_ pH 7.5).

#### Determination of molecular mass

The purified protein was denatured with Laemmli buffer at 95 °C for 5 minutes. The molecular mass of the denatured protein was determined by sodium dodecyl sulfate-polyacrylamide gel electrophoresis (SDS-PAGE). The protein was stained with Coomassie brilliant blue G-250. The molecular mass of the protein was estimated using a protein marker (ThermoScientific, USA) as standard.

#### Autoproteolysis assays

Purified full length protein (0.5 mg/mL) was incubated at different temperatures −21 °C, 4 °C and room temperature (25 °C) - with and without SigmaFAST™ Protease Inhibitor cocktail (Sigma-aldrich, USA). Protein (0.5 mg/mL) was also incubated in different buffers ranging from pH 4 to 8.5 85 at 25 °C. Purified D13 and D34 were incubated at 4 °C and 25 °C. Autoproteolysis of the protein was analyzed on a 12% SDS-PAGE.

#### Peptidoglycan hydrolase activity assay

*Micrococcus luteus* cell wall suspension (Sigma-aldrich, USA) with optical density at 450 nm (OD_450nm_) of 0.62 (0.70 mg/mL) in MilliQ water was incubated in triplicate for 20 minutes at 30 °C (i) Without protein (Buffer: 50 mM NaH_2_PO_4_, pH 5.0) (ii) With PCP (full length) in 50 mM NaH_2_PO_4_ (pH 5.0) 2 μM (0.035 mg/mL). Reading was obtained every minute with prior shaking.

#### Dynamic light scattering

The particle size of the purified protein 13.19 μM (0.5 mg/mL), dialyzed with 150 mM NaCl and 25 mM NaH_2_PO_4_ buffer at pH 8.5 and 25 °C, was determined in a glass cuvette using the molecular size analyzer Zetasizer Nano ZS (Malvern, UK). The system was setup for three runs calculation.

#### Fluorescence spectroscopy

The microenvironment of tryptophan upon ampicillin binding and the binding constant of protein in complex with ampicillin were analyzed by fluorescence quenching spectroscopy. Fluorescence measurements were performed at 25 °C using 50 mM NaH_2_PO_4_ buffer containing 300 mM NaCl and 200 mM imidazole at pH 8.5 in a Jasco FP-6500 spectrofluorimeter (Jasco, Japan) coupled to a Peltier system Jasco ETC-273T with water circulation. Both excitation and emission slits were fitted to 5.0 nm and the excitation and emission wavelength were 295 nm and 300–400 nm, respectively. The concentration of protein was 5.28 μM (0.2 mg/mL), whereas the concentration of ampicillin varied from 0.0029 μM to 57.0 μM. The average of three fluorescence spectra were recorded and processed with the software “Spectra Manager” (Jasco, Japan). Fluorescence intensity and displacement of the corresponding emission band of tryptophan residue of increasing ampicillin concentrations were recorded and fitted according to the classic Stern-Volmer equation (Eq. 1). In order to calculate the binding constant of the protein-ampicillin complex, the equilibrium between free (B_o_) and bounded (B) protein is assumed to be proportional to the fluorescence intensity (F), as [B]/[B_o_] ∝ F / F_o_ and that there are (*n*) binding sites for quenchers (Q) on protein. The binding constant for the equilibrium between free and bound ampicillin to a set of equivalent sites on protein was calculated according to the double logarithm regression (Eq. 2)^86^,^87^:


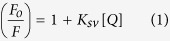



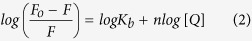


where F_o_ and F are fluorescence intensities in the absence and presence of quenchers respectively, [Q] is the quencher (ampicillin) concentration, *Ksv* is the Stern-Volmer constant, *Kb* is the binding constant and *n* is the number of binding sites per protein.

#### Circular dichroism

The protein secondary structure content, protein folding and protein thermal stability were investigated by circular dichroism spectroscopy. The measurements were carried out using the Jasco J-815 spectropolarimeter (Jasco Analytical Instruments, Japan) equipped with a Peltier type temperature controller (Jasco Analytical Instruments). Far-UV spectra were recorded using a 0.1 cm path length quartz cuvette. Three consecutive measurements were performed in buffer of 2 mM Tris-HCl with pH 8.5, at 25 °C, using protein concentration of 5.28 μM (0.20 mg/mL), and the average spectrum was recorded and corrected to exclude the baseline contribution of the buffer. The protein stability assay was performed by recording the CD spectra at temperatures ranging from 25 °C to 95 °C. The secondary structure content was estimated using the CDNN deconvolution software (Version 2.1). Secondary structure and thermal stability of the protein were also determined in the presence of 2 mM DTT.

#### Small angle X-ray scattering (SAXS) and size-exclusion chromatography with multi-angle light scattering (SEC-MALS)

Small angle X-ray scattering experiments were performed at the bending magnet beamline B21 at the Diamond Light Source synchrotron (Didcot, U.K.). The X-ray wavelength and sample-to-detector distance were 1 Å and 3.9 m, respectively, corresponding to an accessible q-range of 0.004 to 0.4 Å^−1^. SAXS measurements were performed using a specialized size-exclusion chromatography (SEC) configuration that enabled precise capture of the elution peak in a 17 μL flowcell (1.6 mm path length). SEC was achieved with an Agilent 1200 series HPLC and a Shodex silica resin KW403-4F (4.8 mL) column using a running buffer composed of 150 mM NaCl, 25 mM NaH_2_PO_4_, 1% sucrose and 2 mM TCEP. Samples were injected at 10 mg/ml. SAXS measurements were made using a set of 60 10-second exposure frames for a total exposure time of 10 minutes at 15 °C. SAXS data were normalized to beamstop diode readings and integrated using in-house software. Radiation induced aggregation was monitored by comparing the first exposure frame to subsequent frames. Datasets were reduced and processed using Scatter^88^. A sample from the same vial used for the SAXS experiment was used in SEC-MALS analysis to evaluate the homogeneity and molecular weight of the purified protein. An 18-multi-angle light scattering instrument from Wyatt Technology Corporation was used for the measurements.

## Additional Information

**How to cite this article**: Faheem, M. *et al*. Functional and structural characterization of a novel putative cysteine protease cell wall-modifying multi-domain enzyme selected from a microbial metagenome. *Sci. Rep.* **6**, 38031; doi: 10.1038/srep38031 (2016).

**Publisher's note:** Springer Nature remains neutral with regard to jurisdictional claims in published maps and institutional affiliations.

## Supplementary Material

Supplementary Information

## Figures and Tables

**Table 1:** Secondary structure contents of PCP at different temperatures in the presence and absence of 2 mM DTT, estimated from circular dichroism measurements.

Secondary Structure (%)	without DTT		with DTT	
	25 °C	95 °C	25 °C	95 °C
α-helix	26.2	19.8	8.1	4.6
β-antiparallel	15.5	19.0	12.0	46.7
β-parallel	5.7	5.6	19.0	5.4
β-turn	18.3	18.3	23.2	20.3
Random coil	33.0	32.9	45.7	37.3

The secondary structures percentages were calculated using the CDNN deconvolution software^45^.
