# Supplementary material for: Functional and structural characterization of a novel putative cysteine protease cell wall-modifying multi-domain enzyme selected from a microbial metagenome
Source: Sci Rep. 2016 Dec 9;6:38031. doi: 10.1038/srep38031 (PMC5146660; doi:10.1038/srep38031)
Supplement: Supplementary Information [file srep38031-s1.pdf]

Functional and structural characterization of a novel putative cysteine protease cell wall-modifying multi-domain enzyme selected from a microbial metagenome

Muhammad Faheem[1,2], Diogo Martins-de-Sa[1], Julia F. D. Vidal[1], Alice C. M. Álvares[1], José Brandão-Neto[3], Louise E. Bird[4], Mark D. Tully[3], Frank von Delft[3], Betulia M. Souto[5], Betania F. Quirino[2,5], Sonia M. Freitas[1], João Alexandre R. G. Barbosa[1,2,*]

[1]Laboratório de Biofísica Molecular, Departamento de Biologia Celular, Universidade de Brasília, Brasília, DF, 70910-900, Brazil
[2]Programa de Pós Graduação em Ciências Genômicas e Biotecnologia, Universidade Católica de Brasília, Brasília, DF, Brazil
[3]Diamond Light Source Ltd, Harwell Science and Innovation Campus, Didcot, OX11 0QX, England
[4]OPPF-UK, Research Complex at Harwell, Rutherford Appleton Laboratory, Oxford, OX11 0FA, United Kingdom
[5]Embrapa Agroenergia, Parque Estação Biológica - PqEB s/n°, Brasília, DF, 70770-901, Brazil
*joao.argb@gmail.com

Supplementary information

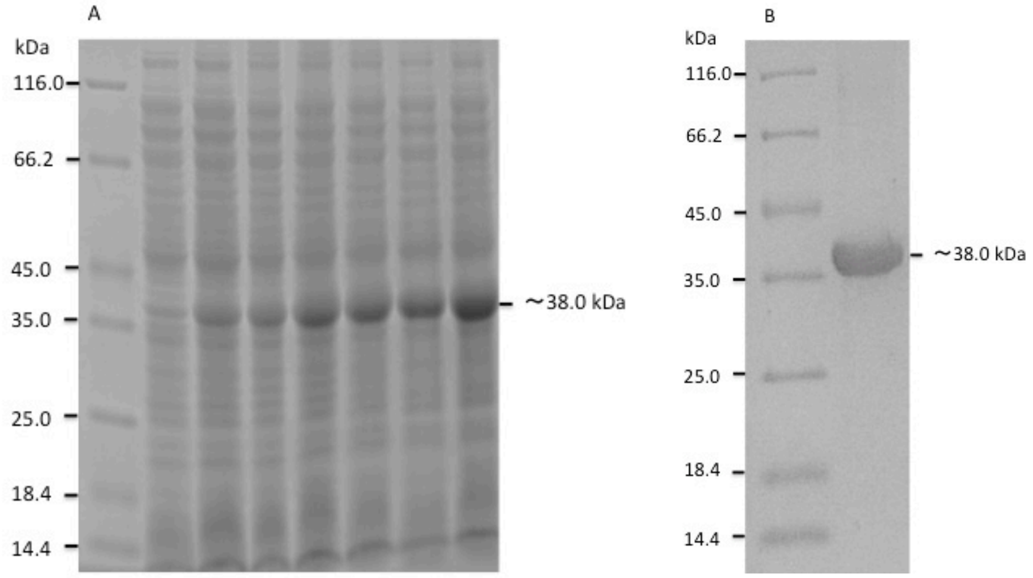

Supplementary Figure 1 (S1). 12% SDS PAGE of the recombinant PCP (indicated as the ~38 kDa protein) expression profile. (A) L1: Protein marker; L2: Pre-induced total bacterial cell protein (TCP); L3: 30mins Post-induction TCP, L4-L8: TCP at 1 h intervals post-induction up to 6 h. (B) L1: Protein marker; L2: Purified protein PCP.

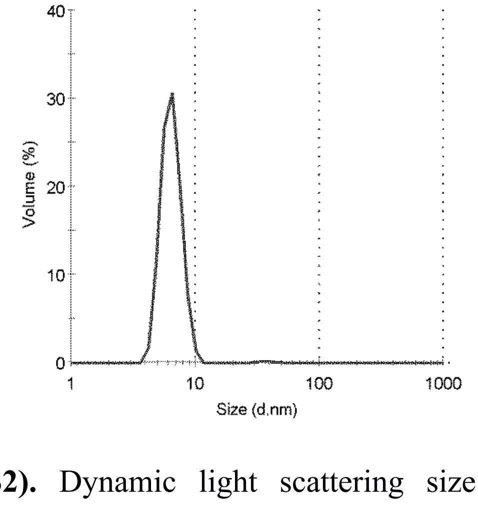

Supplementary Figure 2 (S2). Dynamic light scattering size distribution (hydrodynamics diameter) of PCP.

Size Exclusion Chromatography with Multi-Angle Light Scattering (SEC-MALS) analysis

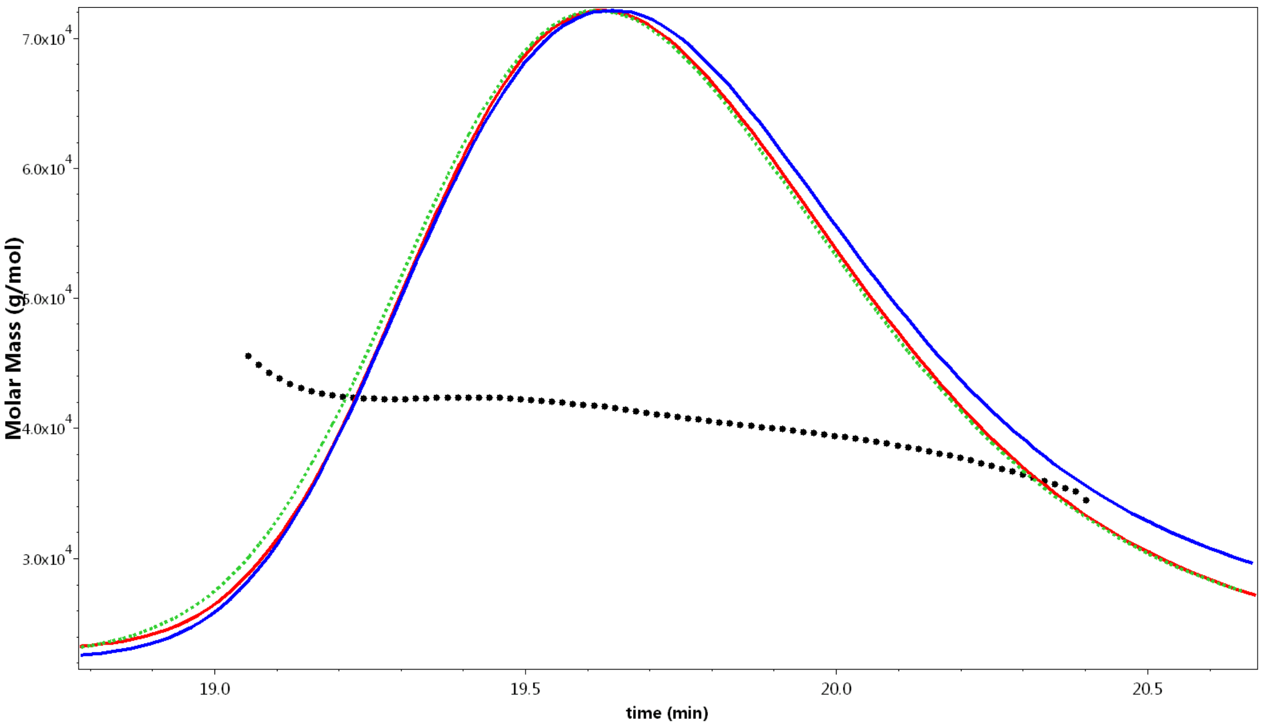

Supplementary Figure 3 (S3). SEC-MALS analysis of PCP protein. The SEC peak for PCP is polydisperse with a spread of molecular weight ranging from 39.8 to 42.3 kDa across the peak. Trace legend: red, light scattering; blue, refractive index; green, UV.

Sequence analysis and homology modeling

Finding domain 1

Interpro and ThreadDom domain prediction indicates that PCP has three domains. However, when each domain was submitted individually to LOMETS server, the threading alignments presented very low homology between the first 48 residues of domain 1 and the PDB templates. The best templates clearly aligned to conserved regions, especially those predicted by MEROPS to be near the catalytic residues. One of these templates, RipA (PDB accession 3NE0), presents a small N-terminal domain tightly bound to the NlpC/P60 domain, which was shown to physically block the catalytic site cleft. While this small domain, belonging to the PB015164 family, has no homology to the N-terminal region of domain 1 from PCP, we hypothesized that the 48 unaligned residues comprised another sub-domain. This was confirmed after a BLAST search was performed with the N-terminal region. At least seven proteins (e-values < 5e-04) have homologues of the N-terminal region at the C-terminus of NlpC/P60 domains (instead of the N-terminus), corroborating that this

region is a distinct domain (Fig. 3A). When submitted individually to LOMETS server, this sub-domain presented 20% identity to the LCI domain from *Bacillus subtilis*, an antimicrobial protein (PDB accession 2B9K), and 26% identity to the C-terminal region of a putative sensor histidine kinase domain (SHK) from *Clostridium symbiosum* (PDB accesion 3FN2). Remarkably, despite only 16% identity between each other, the LCI domain and C-terminal region of SHK have a very similar fold (Fig. S4), with a β-sheet composed of three antiparallel strands in a $\beta_1\beta_3\beta_2$ topology. The only difference is the presence of a small fourth strand ($\beta_{1/2}$) in the loop between β1 and β2 in the LCI domain (Fig. S4). Nonetheless, we termed this fold as the LCI fold considering that the LCI domain is the smallest structure in the PDB presenting this fold. We conclude that PCP, in fact, has four protein domains, with the "new" domain 1 comprising residues 1 through 48.

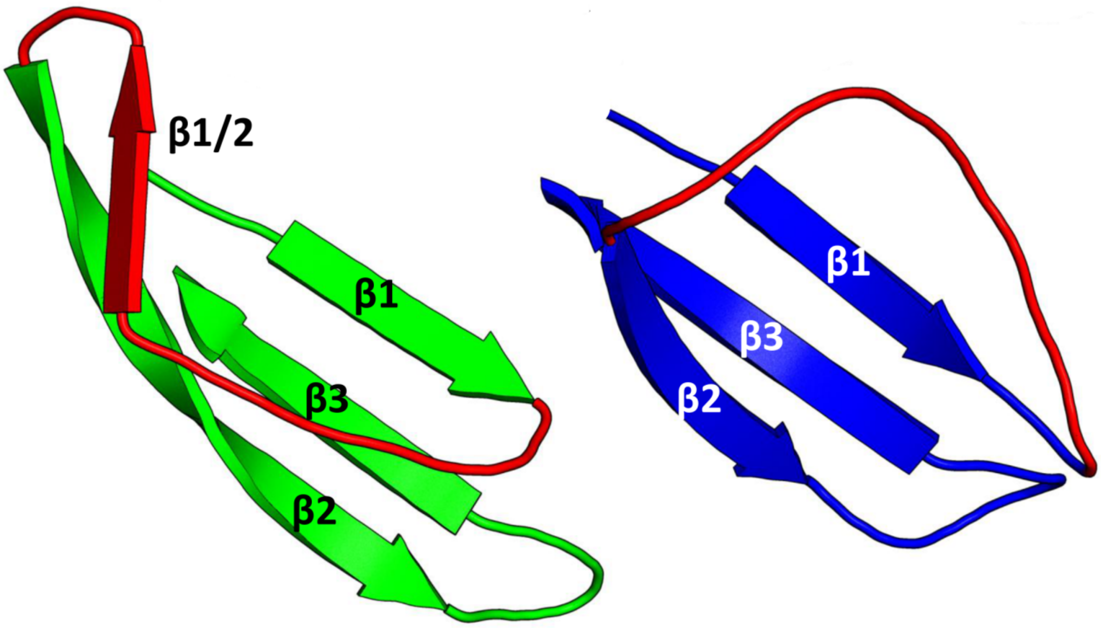

Supplementary Figure 4 (S4). Structure of domain 1 templates. The LCI domain is shown in green (left) and the SHK domain is shown in blue (representing the C-terminal, to the right) and in white (representing the N-terminal, at the upper position). The main difference is the presence of a small fourth strand ($\beta_{1/2}$) in the loop between β1 and β2 in the LCI domain (shown in red). To facilitate visualization, the loop representations were manipulated using PyMOL's "smooth loops" setting. The LCI red loop is 13 amino-acids-long and the SHK red loop is 16 amino-acids-long.

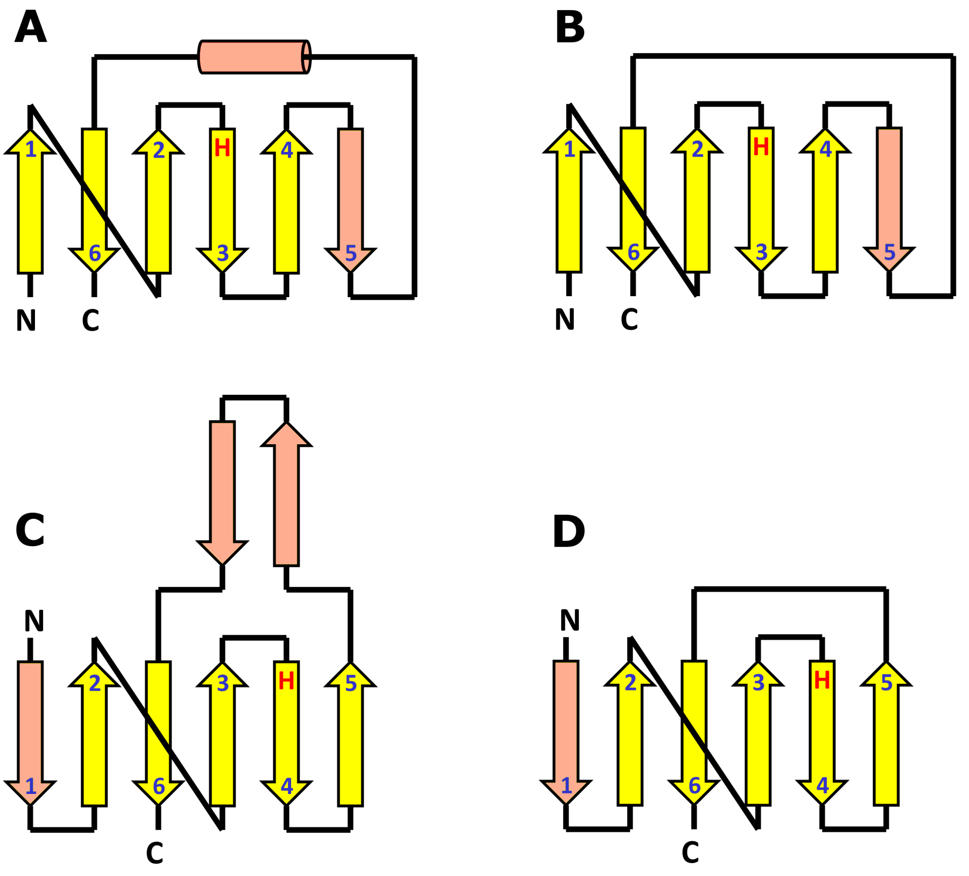

Supplementary Figure 5 (S5). β-sheet topologies in CHAP domains. Two types of topologies can be found for β-sheets in CHAP domains: $\beta_1\beta_2\beta_6\beta_3\beta_4\beta_5$ and $\beta_1\beta_6\beta_2\beta_3\beta_4\beta_5$. (A) Topology found in PDB structures 4HZ9, 2EVR, 2HBW and 4XCM. (B) Topology found in PDB structures 3I86, 2XIV, 3NE0 and 3PBI. (C) Topology found in PDB structure 3A2Y. (D) Predicted and modeled topology for PCP. For each topology, the catalytic histidine is depicted in red and the conserved arrangement of β-strands are emphasized in yellow.

Interdomain interactions

When we analyze D1 and D2 in PCP homologues that have an upstream domain 1 in regards to domain 2, it is possible to observe some indications of adaptive evolution between residue pairs. The basic hypothesis connecting correlated substitution patterns and residue–residue contacts is very simple: if two residues of a protein or a pair of interacting proteins form a contact, a destabilizing amino acid substitution at one position is expected to be compensated by a substitution at the other position over the evolutionary timescale, in order for the residue pair to maintain attractive interaction[1]. The most prominent of these pairs is Arg45/Asp152. This pair is either completely conserved (32 out of 41 sequences) or completely mutated (9 out 41 sequences). Example of the mutated Arg45/Asp152 pair can be seen in (Fig. S6). This observation suggests that this residue pair might be involved in the formation of salt bridge between domain 1 and domain 2.

Also, several hydrophobic groups can be identified in the PCP sequence (i.e. regions presenting ≥ 50% hydrophobic amino acids). These groups comprise amino acids 1-10, 29-44, 50-70, 76-87, 102-118, 125-141, 147-155 and 163-166. Upon tertiary structure analysis, these groups reveal hydrophobic patches that can either be structural (buried) or superficial (solvent-exposed). Superficial hydrophobic patches can indicate where interdomain interfaces are likely to form, as well as where ligand and substrate may bind. This might be the case of the hydrophobic clump comprising residues 50-70, which was predicted to interact with domain 3 in SAXS fit.

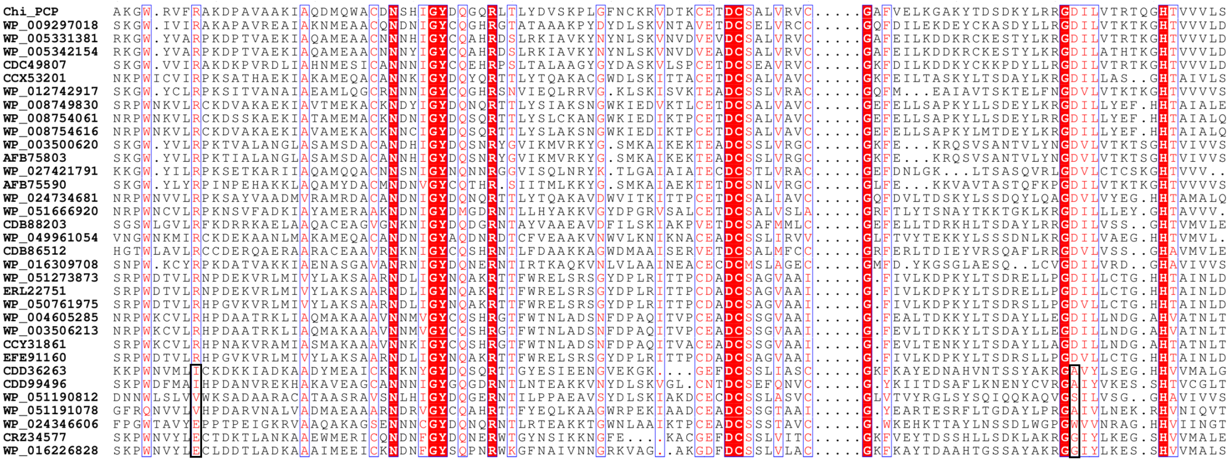

Supplementary Figure 6 (S6). Alignment of domains 1 and 2 regions with homologous sequences. Concomitantly mutated Arg45 and Asp152 residues are depicted in the black boxes and suggest possible interaction sites between domain 1 and domain 2.

Supplementary Table S1 – Comparison of secondary structure content determined in CD and homology modeling. Values are shown as percentages of the total.

Secondary Structure	CD	Homology model
α-helix	26.2	23.8
β-sheet	21.2	22.3
Other	51.3	53.9

Supplementary References

1 Morcos, F. *et al.* Direct-coupling analysis of residue coevolution captures native contacts across many protein families. *Proceedings of the National Academy of Sciences* **108**, E1293-E1301 (2011).
